# The Planispheric Optic Array

**DOI:** 10.1177/2041669517705388

**Published:** 2017-05-05

**Authors:** Jan Koenderink, Andrea van Doorn

**Affiliations:** University of Leuven, Belgium; Utrecht University, The Netherlands

**Keywords:** panoramic vision, visual space, pictorial space

## Abstract

The “planispheric optic array” is a full-horizon Mercator projection of the optic array. Such pictures of the environment are coming in common use with the availability of cheap full-view cameras of reasonable quality. This introduces the question of whether the public will actually profit from such pictorial information in terms of an understanding of the spatial layout of the depicted scene. Test images include four persons located at the corners of a square centered at the camera. The persons point at each other in various combinations. Participants in the experiment judge who is pointing at whom in a number of such photographs. It is found that certain very systematic and huge errors are the rule, indicating that naïve viewers are quite unable to parse such planispheric representations.

## Introduction

Full optic array (Burton, 1945; Gibson, 1950; Koenderink et al., 2010; Reid, 1819) panoramic cameras are becoming increasingly affordable, easy to use, and thus popular. They do indeed offer some singular advantages. For instance, it is not needed to point the camera anywhere, and one obtains a full record of the environment in a single shot. Such properties make it an attractive note-taking gadget for many purposes, including family holidays. However, there are also disadvantages; for instance, the fact that every photograph is necessarily a selfie and that the change of pictorial size with distance becomes a little too much explicit to most persons’ taste.

There are other, at first blush, less obvious disadvantages though. The one focussed upon here is the inability of visual awareness to deal with most pictorial representations of the results (Attneave & Farrar, 1977; Koenderink, van Doorn, de Ridder, & Oomes, 2010; Koenderink, van Doorn, & Lappin, 2003; Phillips & Voshell, 2009). These are of two major categories, static images and images under the viewer’s control. An example of the latter kind are images in which the observer may pan around the horizon, as is easily implemented in smartphone apps. (Please try the videos provided with this article on the publisher’s website—they should be run in loop mode.) In this article, the emphasis is on static images, although the results allow inferences that apply to certain dynamic cases as well.

The most popular static representations are cylindrical projections with the horizon represented as a (straight) horizontal line. Various cylindrical projections are in common use. The camera usually delivers a so-called *flat map* (or equirectangular projection, G. *Plattkarte*, Fr. *plate carrée*), with azimuth and elevation as Cartesian coordinates. Such a map has aspect ratio two (ratio of the arc length of the equator to that of a meridian) and is strongly deformed near the zenith and nadir of the optic array. This map is quite acceptable if it is suitably confined though (Hauck, 1879; Koenderink, van Doorn, Pinna, & Pepperell, 2016). This basic camera data structure can of course be transformed into any of a great many *projections*. In this experiment, the Mercator conformal map is used, hence the term *planispheric*: Gerardus Mercator (1512–1594) published his famous map (Mercator, 1569) as a planisphere depicting the earth. The conformal property is nice whereas global deformations are limited if the elevations are not too large. It is a useful projection that is very similar to what is already in common use. When deviations from the horizon are limited, all cylindrical projections are essentially interchangeable.

A cylindrical full-horizon rendering is a rectangular picture. The aspect ratio depends on the range of elevations and on the type of projection. Since it was decided to focus on conformal mapping (no local deformations), the type had to be Mercator. In this study, the aspect ratio was set to the Golden Ratio (i.e., $\phi =(1+\sqrt{5})/2=1.61803\ldots$, or 144/89 with an error less than 1%), for no better reason than that it basically looks like a postcard. This limits the elevations to ±gd(π/ϕ)≈73.6707…deg (here gd(x) denotes the Gudermannian function [Cayley, 1862]). So we skip about a 16deg—radius zenithal cap (same at the nadir, of course).

The map is conformal, thus locally a similarity. The global deformation occurs as a change of local magnification from unity at the horizon to infinite at the zenith and nadir. Due to the omission of the polar caps, the maximum magnification in our images is cosh(π/ϕ)≈3.557…. It is hardly noticeable because of the irrelevant subject matter near the lower and upper edges of the image. At 25% and 75% height in the image, the magnification is only about 1.51.

Due to the removal of the polar caps, the panorama subtends about 360deg×147deg, which is *huge* by conventional standards. The eye can hardly see more than 180deg horizontally or 150deg vertically without head and body movements (Polyak, 1941), typically this would involve many fixations. Thus the picture offers a data structure that is very unlike any view anyone ever had, although it (at first blush) appears as a regular postcard. In view of this major mismatch, it is reasonable to expect problems in parsing this data structure. However, most naïve observers take it in their stride, after all, it certainly *looks* like a regular postcard. Small wonder then that this gives rise to major misinterpretations.

Visual awareness is simply unable to deal with it. No doubt, most people can be trained to veridically interpret such images. One should reveal to them that the correct response to certain two-dimensional pictorial structures is such and so. Memorizing a simple, short list will do. But such a veridical interpretation is likely to be essentially unrelated to what one is visually aware of. Immediate visual awareness cannot be influenced by formal knowledge as is illustrated by such well-known “geometrical illusions” as the Poggendorff, Zöllner, and Müller-Lyer. The errors will remain unnoticed when the exercise of veridical interpretation is skipped, as will be typical for most naïve users. This research is about quantifying such errors. It may well find some use in various application areas.

## Methods

This study investigates judgments of the internal structure of pictorial content by naïve observers confronted with planispheric full-horizon pictures. The judgments concern the visual awareness of environmental spatial relations based on pictorial content. The study should probably be classified as “pictorial perception” albeit in a rather unusual setting.

### Pictures

**Figure 1.**
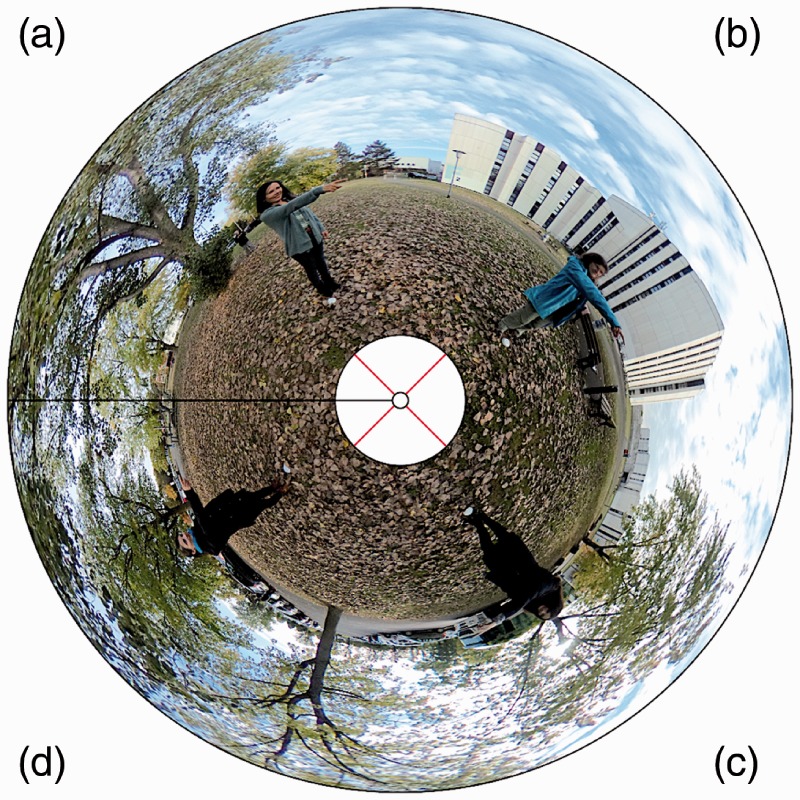
An annular map of Stimulus i (see Figure 4) in postel projection (Riemann normal coordinates) centered at the nadir (so-called *small earth* representation). Polar caps (30degradius) centered at zenith and nadir were deleted, so only an environment of the horizon is shown, which has the topology of an annulus ${\text{S}}^{1}\times {\text{I}}^{1}$ (see the section “A model”). The black line indicates the cut-locus used in the Mercator projection. In the annular representation, it is obvious that the actors are pointing cyclically and the cut-locus is not in effect. The red lines connect mutually antipodal actors.

**Figure 2.**
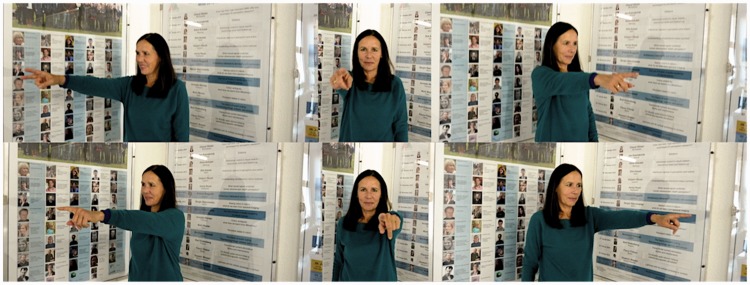
These photographs show the perspective of an actor from the position of the camera. Here the actor was placed in a right-angled corner and pointed either along a wall or the bisectrix of the walls, whereas the camera was placed on the bisectrix. This allows one to judge the perspective, which is the same as in the actual stimuli.

Pictures were taken in an environment that was very familiar to all participants. They all depicted four actors apparently pointing— right arm outstretched horizontally, in the actor’s somatic forward direction, the head facing the direction being pointed at. (The actor’s gaze could not be made out very well in the pictures.) There were four actors involved, they were situated at the corners of a square that was centered at the camera position. The camera was at a height of 150 cm, the distance of each actor to the camera was also 150 cm. The setup is shown in Figure 1 in an annular projection. Figure 2 illustrates the pointing geometry.

**Figure A1.**
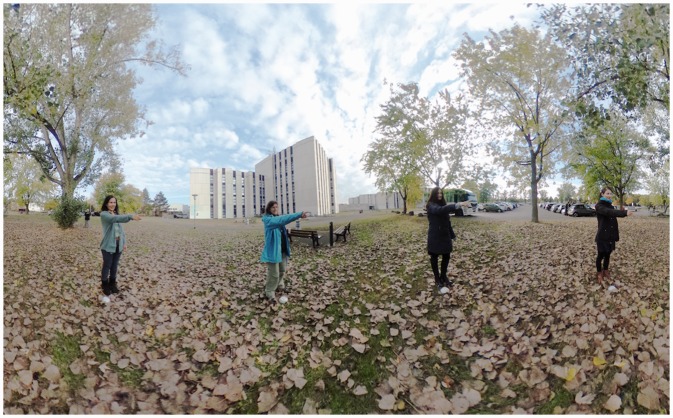
Stimulus i.

The camera was a Ricoh Theta S (Ricoh, 2016), which is a small package using a sandwich of two back-to-back fish eye lenses, each with a field of view of 190deg. The two images are seamlessly combined in the camera. The camera can be remote controlled via an iPhone over a WiFi link, this was exploited so as to avoid including the photographer prominently in the images. The camera was fixed during the session, so were the locations of the actors. However, the actors went cyclically through the positions, so their position with respect to the background is variable (see Appendix Figures A1 through A7).

Although this setup may sound simple enough, one meets with a combinatorial explosion. For whereas each actor might point to three others, an actor might also not be pointing at all, or may point out of the scene. Including all possibilities in the task yields an unmanageably large number of possible stimulus images. Yet it is tantamount to the validity of the experiment that this number be severely restricted. The reason is that participants should spend not more than a few minutes or so on the task in order to be reckoned naïve. Given time, they might conceivably use non-perceptual reasoning to cognitively “compute” a response instead of relying on their actual visual awareness. The eventual success of the present choice is checked in the results.

**Figure 3.**
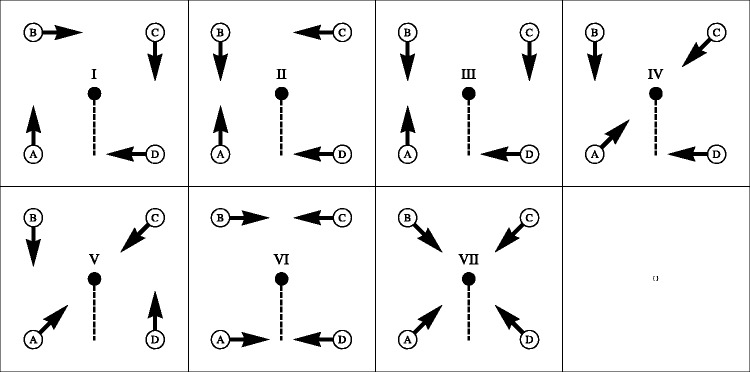
Ground truth for all cases (i through vii). The camera is located at the black dot. The edges of the planispheric representation are defined by the direction of the dashed line, the full horizon (360deg) being represented. Actors are located at the locations A, B, C, and D pointing in the directions of the arrows. Although they point with outstretched arm and extended index finger, they also assume postures such that body and head face the target.

**Figure 4.**
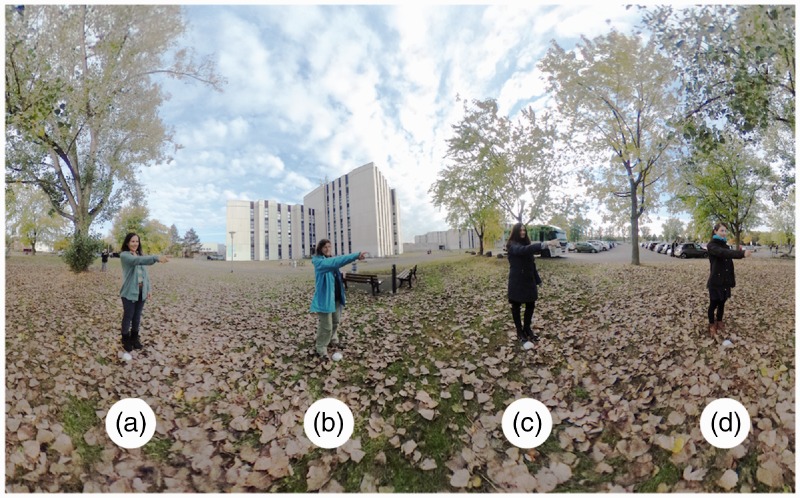
Same photograph as shown in Figure 1 but in the planispheric projection used in the experiment. This shows less than the full optic array. Although the horizon (horizontal midline) is fully represented, the elevations only involve the range ±74deg. Notice the symmetrical placement of the actors. The background is very familiar to the participants and the same in all photographs. The actors cycle positions though.

The final compromise involves seven photographs, with all actors always pointing to some other actor. Even with this restriction, seven photographs do not exhaust the possibilities. The actual choice is illustrated in Figure 3. An example image is shown in Figure 4. All stimuli can be seen in the Appendix Figures A1 through A7.

In retrospect, this selection proves indeed sufficient to permit strong conclusions. However, it certainly leaves many interesting questions undecided.

### Presentation and Observers

**Figure A8.**
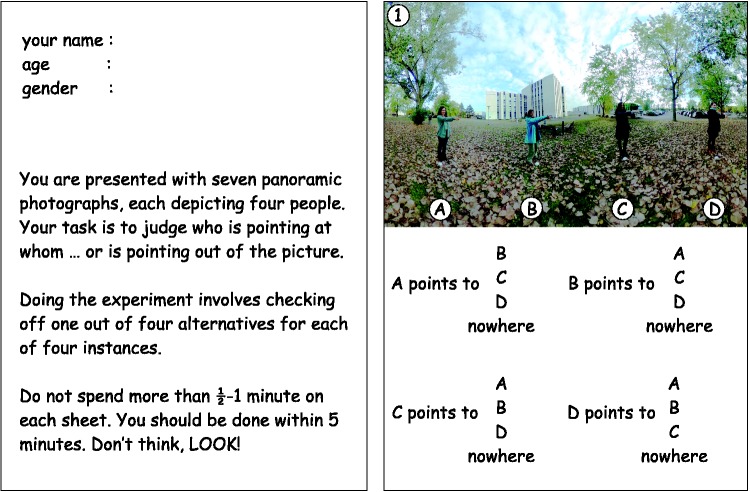
Cover sheet and Sheet 1.

The photographs were presented as printouts, each photograph filling the top-half of a sheet of A4 paper. The bottom of the sheet was used for marking judgments. Participants were handed a pile of seven sheets (the seven photographs) with an additional cover sheet containing instructions. (See Appendix Figure A8, left and right.) They went through the questionnaire (if one may call it that) quite fast and in the presence of an experimenter (who kept silent during the marking).

There were 25 participants, each marking all seven cases, each case involving four questions (like “A points to ?”), each question admitting of four (B, C, D, or nowhere) answers. Thus the total data volume collected subtends 1,400 bits of information (=25×7×4×log24).

Observers were PhD students, postdocs, and staff from the department of psychology of Giessen University. Ages varied widely, median 30, interquartile range 27 to 38. Distribution over genders was about even (40% female).

The actors that figure in the stimuli did not participate as observers.

## Experiment

Participants completed their questionnaires within a few minutes in an informal setting, usually their own desk. Viewing was not restricted. Participants used normal viewing distance, good lighting, wore their usual correction, and so forth. This was done in order to ensure that the results would be representative for the generic viewing of pictures in daily life situations. Participants were asked to trust their visual awareness, rather than reasoning out the geometry.

The raw data from the completed questionnaires consist of 175 lines, each line containing three types of data, the identification of the participant, the identification of the case, and the four responses. Participants are identified by Index 1 to 25, age, and gender. The case is identified by an Index 1 to 7, referring to the cases that will be denoted as “case iv”, and so forth, in this article. The responses are coded by an ordered set of capitals “A,” “B,” “C,” “D,” and “N,” where A to D denote locations whereas “N” denotes “not pointing to any of the actors.” Thus a response NANC is read as “A points to no one, B points to A, C points to no one and D points to C.”

The various analyses mainly involve counting various cases in this dataset.

## Analysis

A first analysis aims at a global impression of the coherence of the data. This involves two aspects, first to what degree participants can perform the task and second how different participants compare in their results.

**Figure 5.**
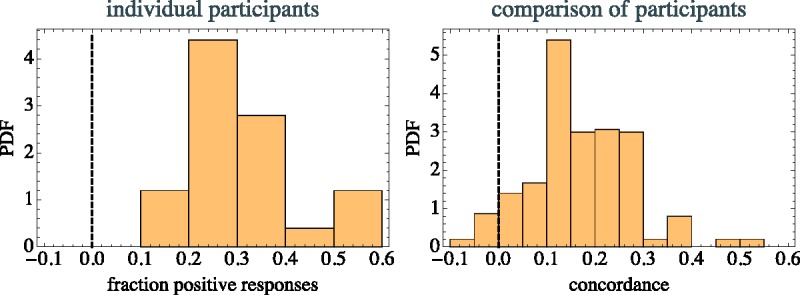
The probability density of the fractions of positive responses per participant (left) and that of the concordances (right).

As to the first aspect, one way to judge this is to simply find the fraction of positive responses, that are responses that indicate a location A to D instead of nowhere (N). The median of the fraction is 0.29 (interquartile range: 0.25–0.36), full range spanning 0.1 to 0.5 (see Figure 5, left). Although there is quite a bit of variation, it is evident that all participants are ready to perform the task to some degree. The fact that roughly three quarters of the responses are not to any of the actors (N) indicates that the task is apparently not as straightforward as might have been expected.

As a measure of concordance between observers, one needs to consider their responses to the same instances. The measure used was to rate a comparison as zero if at least one of the responses was N, as +1 if they were equal but not N, and −1 if they were unequal and neither of them N. (Notice that the pair {N,N} thus does not count as a concordance!) The concordance is the mean of all of these contributions. It apparently ranges between −1 and +1. The aforementioned fraction of positive responses is simply the concordance of a participant with that participant itself.

**Figure 6.**
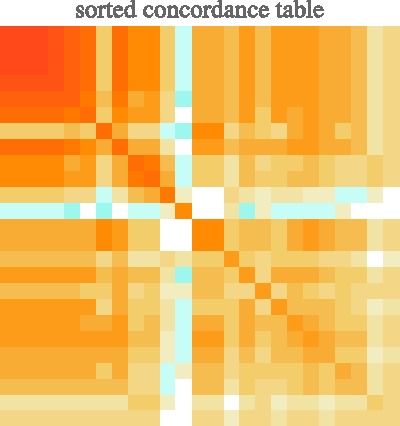
The concordance table is obviously symmetric but here plotted in full for easier visual reference. The participants have been sorted by their self-concordance (highest at top-left). Reddish colors indicate positive, bluish values negative concordance. Two or three “dissenters” are immediately visible in the plot. Notice that concordance tends to be higher for participants with high positive fractions.

The median concordance is 0.18 (interquartile range: 0.11–0.21), full range −0.07 to +0.5 (Figure 5, right). Thus the concordances are quite high. They tend to be larger between observers with a high positive fraction. There are apparently two or three participants that might be classified as “dissenters” (Figure 6). It was decided to leave them in the total dataset instead of omitting them from the analysis. Since they subtend perhaps 10% of the participants it is hardly of interest to investigate the dissenters in detail.

A next global analysis involves the overall distribution of correct responses, errors, and misses. Here a *miss* is an “N” response. It is indeed a miss because in each instance there is a veridical answer among A to D. Correct responses and errors are identified through comparison with the ground truth. Thus an *error* is defined as a response indicating the wrong actor. There are 29% correct responses, 2% errors, and 69% misses overall. As remarked above, the fraction of misses is quite high. What is of main interest here is that the fraction of errors is very low, much lower than the fraction of correct responses. The fraction of errors among the positive responses is only about 7%.

**Figure 7.**
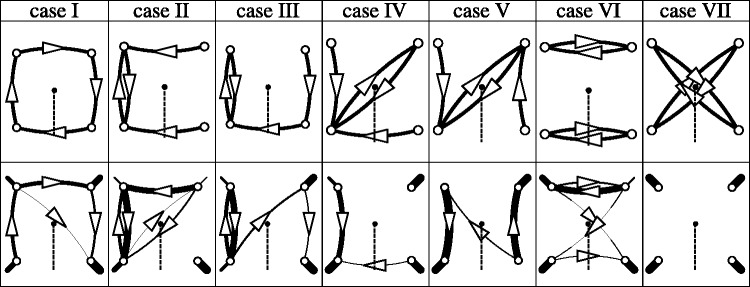
Results for the separate Cases i–vii. At the top row, the ground truth, a drawn edge indicating the presence of a pointing relation between locations. At the bottom row, the compiled counts. Here the thickness of a drawn edge is proportional with its count in the total number of responses for the case. The “N”—responses have been indicated by short lines pointing away from the center of the square. (There are no “N”—instances in the ground truth.)

A more differentiated view of the data is obtained by doing the analysis per Case i to vii. This is perhaps most clearly seen in a graphical representation that allows immediate comparison of the results with the ground truth (Figure 7).

Immediately striking are two facts:

^ Positive responses involving the outermost actors in the pictures are extremely rare. Hardly ever gets a participant the pointing wrong, but pointing involving both A and D usually results in misses.
  ^ No positive responses are recorded involving the pairs {A, C} or {D, B}.

Both instances account for the bulk of the misses. The graphical representation is so clear that it hardly serves a purpose to list explicit counts.

What *is* of interest is to look into more detail for some of these instances, for it turns out to be the case that especially the errors are very systematic and perhaps surprising.

All errors involve in-picture pointings. The four cases that occur are as follows:

^ In the case of the ground truth A→B, the participant reports A→C.
^ In the case of the ground truth B→C, the participant reports B→D.
^ In the case of the ground truth C→B, the participant reports C→A.
^ In the case of the ground truth D→C, the participant reports D→B.

**Figure 8.**
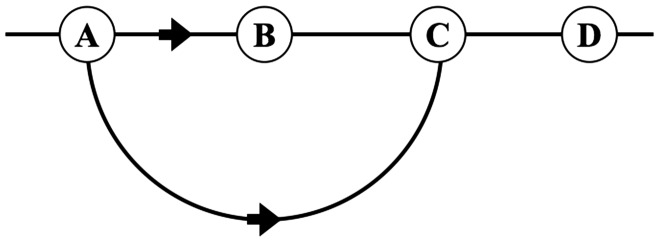
This is the case of “over-pointing” in the visual representation. Whereas A *should* point to B, A is reported to point to C. In view of the pointing direction in the visual representation, this is perhaps less surprising. In Figure 4, one sees that the actors do not appear to point in the picture plane but somewhat toward the observer too. This is due to the fact that the angle between the local camera axis and the pointing arm is $45^{\circ }$, rather than $90^{\circ }$ (Figures 2 and 3). Some observers tacitly take this into account, even though they do not understand the layout of the physical scene.

That is to say, the participant fails to respond to the (actually correct) nearest neighbor, but points to the next more distant one, in those instances where the *over-pointing* can take place in the picture (see Figure 8).

This sums up the remarkable findings. Of course, there are minor additional observations to be made, but these occur in such small numbers that they might be dismissed as due to trivial causes.

## A Model

A reviewer challenged us to explain the data with a model. The major model that accounts for our findings (see Discussion section) is of a topological nature. It involves relations between four structures: the scene in physical space, the projection of the scene on the optic array, the representation of the optic array in the picture, and the scene in visual awareness.

The scene in physical space involves four actors and the camera, all standing on the ground plane, in configurations shown in Figure 3. The camera captures all visual directions.

The optic array has the topological structure of the sphere ${\text{S}}^{2}$. However, only a strip straddling the horizon is used, polar caps at zenith and nadir are discarded. What is left is the product of the horizon (a circle) and a range of elevations (a linear segment), thus ${\text{S}}^{1}\times {\text{I}}^{1}$. This is an annulus, it is illustrated in Figure 1.

The picture is a Mercator map of the annulus. This involves the selection of a cut-locus, such a selection is purely conventional, like the Greenwich meridian (*prime meridian*) on the globe. The Mercator projection maps the annulus on a rectangle, topologically ${\text{I}}^{1}\times {\text{I}}^{1}={\text{I}}^{2}$. The left and right boundaries of the rectangle are both images of the cut-locus. An example is illustrated in Figure 4 (Stimulus i).

We are only concerned with the spatial order of the content of visual awareness when viewing the picture, that is, the Mercator projection. Here, we hypothetically propose two aspects of psychogenesis that are up to empirical test (the *topological model*):

^ The observer treats the picture as a topological rectangle ${\text{I}}^{2}$.
^ The observer is unaware of the relations between the camera and actor positions.

Because the task only involves spatial order along the horizon, one may simplify as follows:

^ The physical scenes involve four points on a circle ${\text{S}}^{1}$ in the Euclidean plane ${\text{E}}^{2}$, with the camera at its center. The camera induces the antipodal relation between points of the circle that are mutually collinear with the camera.
^ The picture is a linear segment ${\text{I}}^{1}$. When the endpoints (boundary) are identified, this reduces to the topology of ${\text{S}}^{1}$. Antipodality is a shift over half the picture width.
^ The spatial order in awareness is a linear segment ${\text{I}}^{1}$ whose end points are distinct. There is no sense of antipodality. (The model assumptions.)

This leads to explicit predictions. Notice that, relative to actor **P** (say), an actor **Q** can be positioned in the clockwise, the anticlockwise, or the antipodal location.

The possibilities are as follows:

**Table:** 

Location	Clockwise	Anticlockwise	Antipodal
A	B	D	C
B	C	A	D
C	D	B	A
D	A	C	B

In the case of the spatial order in visual awareness we note that, relative to actor **P** (say), an actor **Q** can be located to the left or to the right.

The possibilities are as follows:

**Table:** 

Location	Left	Right
A	–	BCD
B	A	CD
C	BA	D
D	CBA	–

Here, the sequence is relevant, thus going from A rightwards to meet first B, then C, finally D. Consequently, when A is pointing to the right, one predicts that the participants would reply that “A points to B” (the first item encountered).

The predictions are confronted with the observations in the next section.

## Discussion

The overall results lead to the following conclusions, the numbers being so evident that no statistics is necessary. Indeed, this is one case where “the data speaks for itself”:

^ The task is hard. In most cases, the participants are not certain about to what other actor an actor is pointing.
^ When a certain positive pointing is reported, it is almost always correct.
^ Pointings that involve diametrically opposite (with respect to the camera position) actors are always missed.
^ Pointings that involve the connectivity of the horizon (the periodic topology in the horizontal direction) are almost always missed.
^ Pointings between actors that are neighbors in the picture are usually reported and if so are mostly reported correctly.
^ In rare cases, pointings within the picture skip a location. This accounts for all the (rare) errors.

Thus both errors and misses are very systematic. The bulk of the data is well predicted by the topological model explained in the section earlier.

The misses evidently come in two types, the ones concerning diametrically opposite locations (let’s call them Type 1 misses) and the ones concerning pointing out of the picture in the horizontal (let’s call them Type 2 misses).

*Type 1 misses* involve the relation of the viewer to the picture. Apparently participants fail to relate diametrically opposite locations, collinearity with the camera is not recognized (our second hypothesis). They cannot relate what is behind their backs to what is in front of them;

*Type 2 misses* evidently result from a failure to recognize—in visual awareness that is— the periodic boundary relation. It involves an incomplete understanding of the topology of the picture frame (our first hypothesis).

Both types of misses are not that surprising, in fact, they are well predicted by the topological model.

The nature of the errors is more mysterious in that the topological model only partly accounts for them. They become somewhat understandable in terms of the structure of visual space as has been discussed in previous articles (Koenderink & van Doorn, 2008).

**Figure 9.**
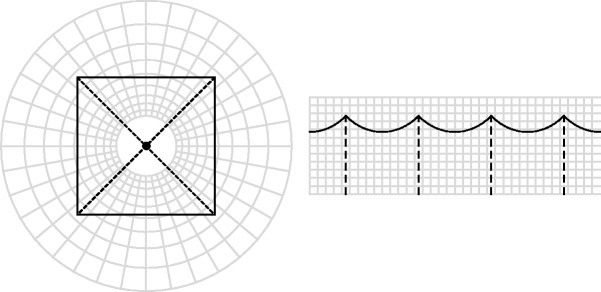
The internal visual representation of pictorial space is probably somewhat like this. The layout in physical space (at left) is mapped on the space of awareness (right). The thick, scalloped curve represents the edges of the square. The dashed rays point to the camera but also to diametrically opposite points in physical space. In the visual representation, they do not point to any of the other actors though, but are mutually parallel.

A representation of visual space is shown in Figure 9. This representation is based on the empirical fact that visual awareness apparently relates spatial attitudes to the local visual direction, instead of an absolute reference frame in physical space (Koenderink, van Doorn, de Ridder, & Oomes, 2010; Koenderink, van Doorn, & Todd, 2009). Thus the bundle of visual directions that all emerge from a perspective center is experienced as a bundle of mutually parallel lines. This puts the perspective center at infinity, that is to say, outside visual space, thus capturing the phenomenological fact that the eye cannot see itself. The formal description is the complex logarithmic map (perhaps familiar from its use in modeling the cortical representation of the retina in V1). In Figure 9, the pointing geometry has been represented in this space.

We have found earlier (Koenderink, van Doorn, de Ridder, & Oomes, 2010; Koenderink et al., 2009) that the representation shown in Figure 9 yields an effective explanation for the errors commonly made in looking at wide-angle photographs. Thus, the description of visual space effectively doubles as a description of pictorial space. In the previous experiments, the scope was limited to about 120deg horizontally, though, which is indeed wide angle but not panoramic as in the present study. Even in wide-angle pictures, observers routinely commit systematic errors of up to 100deg of visual angle. Such errors are quantitatively predicted by the representation of Figure 9. In the present case, the effects are even more pronounced, due to the truly panoramic nature of the pictures used as stimuli.

In this representation, as in visual awareness when looking at the pictures, the actors appear in a frontoparallel plane. Their pointing directions are not in this common plane though, thus the actors appear to point also somewhat in the forward direction. The reason is that their pointing directions in physical space always subtend a45deg angle with the direction to the camera. Perhaps surprisingly, none of the observers remarked that this was a problem. However, the rare erroneous reports become perhaps somewhat more understandable when regarded this way (Figure 8).

## Conclusions

The study involves an exocentric pointing task in pictorial space. There has been research on exocentric pointing in both physical (Koenderink et al., 2003; Koenderink, van Doorn, Kappers, Doumen, & Todd, 2008) and pictorial space (Wagemans, van Doorn, & Koenderink, 2011). However, the present task differs significantly because of the pictorial representation which involves periodical boundary conditions, thus pictorial representation of objects both in front and behind the camera. (Of course, “in front” and “behind” are actually undefined for the camera used here.)

Viewing of static full-horizon pictures in the usual cylindrical projections is not at all intuïtive to the naïve observer. Some of the major problems can be ameliorated by means of interactive presentations though. For instance, offering the observer horizontal (periodic) panning control should take care of most Type 2 misses, at least, given time and opportunity. This can be judged from the video sequences (to be run as loops) provided as additional material (on the publisher’s website) to this article.

The problems with Type 1 misses are less easily solved. Adding additional degrees of freedom in the interaction might help but is unlikely to solve the problem completely. The best bet might be the popular full sphere “rolling ball” rendering. However, this has the disadvantage of placing the observer outside the optic array, so it does not help much for the pointing task. People have little intuition for the geometry of the sphere, as is evident from the common errors made in the estimation of the shortest routes for long-distance flights. Such representations are likely to be more of a hindrance than a help, although most people apparently enjoy them at least for a while.

To use full-horizon pictures effectively, it may be more effective to manipulate the scene in such a way as to fit the structure of pictorial space. For instance, the errors will no doubt be less when the locations and directions in visual space are *off* in certain precise ways (e.g., the demo in [Koenderink, van Doorn, de Ridder, & Oomes, 2010]). However, whereas this may be useful for the purposes of art direction, it hardly applies to the snap-shot culture of the general public.

When using full-panoramic renderings in technical applications, users should be aware to mistrust their immediate visual awareness, to use explicit cognitive reasoning and to use overlaid coordinate grids to best advantage. Using representations that are less intuitively *natural* might help to abstract from immediate visual awareness. From a phenomenological perspective, it may be best to imagine oneself as an external observer of the optic array. Such techniques serve to bring the topic outside the realm of vision science proper.

## Figures and Tables

**Figure A2.**
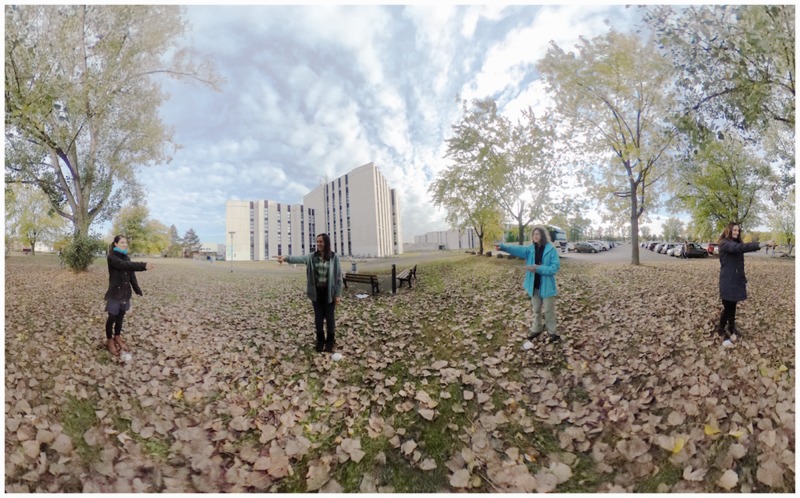
Stimulus ii.

**Figure A3.**
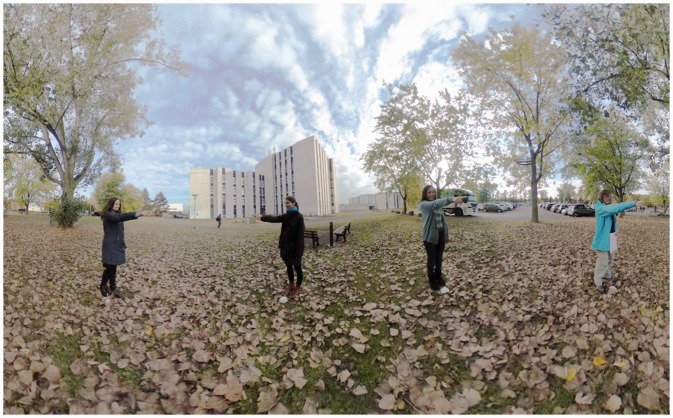
Stimulus iii.

**Figure A4.**
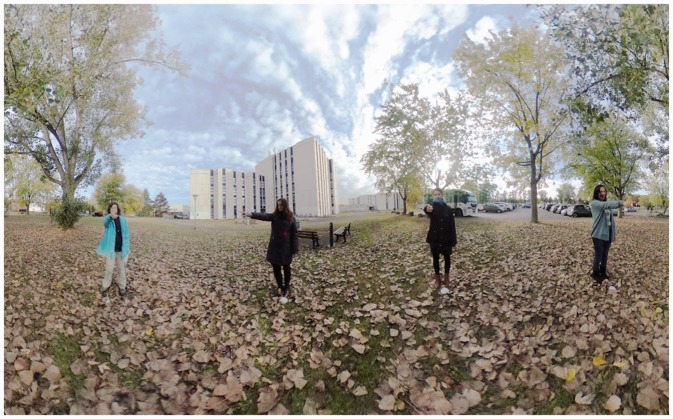
Stimulus iv.

**Figure A5.**
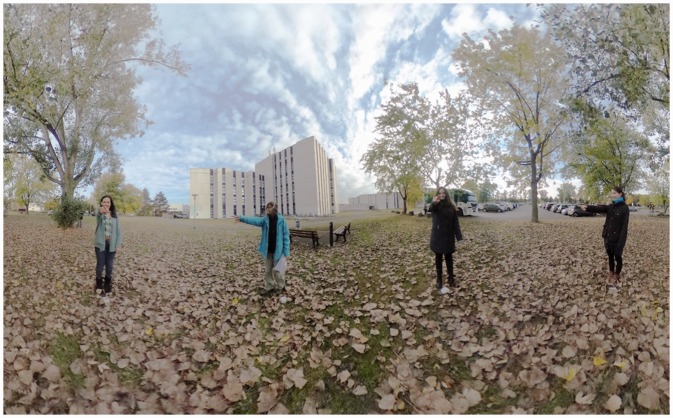
Stimulus v.

**Figure A6.**
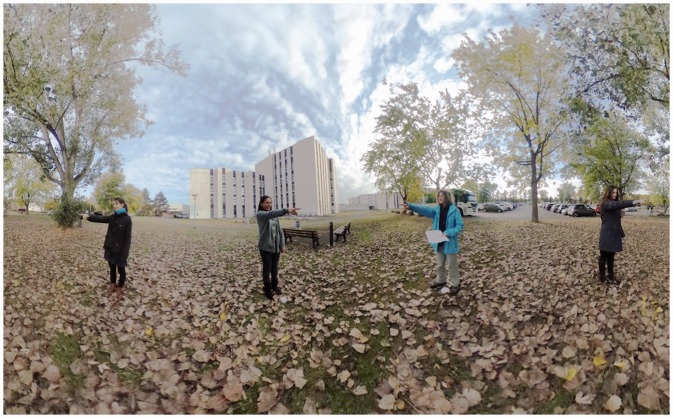
Stimulus vi.

**Figure A7.**
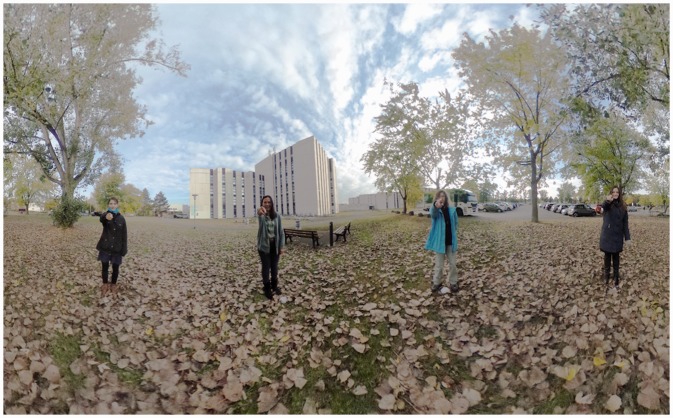
Stimulus vii.

## References

[bibr1-2041669517705388] Attneave F. Farrar P. (1977) The visual world behind the head. American Journal of Psychology 90: 549–563. 610446

[bibr2-2041669517705388] Burton H. E. (1945) The optics of Euclid. Journal of the Optical Society of America 35: 357.

[bibr3-2041669517705388] Cayley A. (1862) On the transcendent gd. u. Philosophical Magazine (4th ser.) 24: 19–21.

[bibr4-2041669517705388] Gibson J. J. (1950) The perception of the visual world, Boston, MA: Houghton Mifflin.

[bibr5-2041669517705388] Hauck, G. (1879). *Die subjektive Perspektive und die horizontalen Curvaturen des dorischen Styls. Eine perspektivisch–ästhetische Studie* [On subjective perspective and the curvatures of horizontals in the doric style. A study on the aesthetics of perspective]. Stuttgart, Germany: Wittwer.

[bibr6-2041669517705388] Koenderink J. van Doorn A. (2008) The structure of visual space. Journal of Mathematical Imaging and Vision 31: 171–187.

[bibr7-2041669517705388] Koenderink J. van Doorn A. de Ridder H. Oomes S. (2010) Visual rays are parallel. Perception 39: 1163–1171. 21125944 10.1068/p6530

[bibr8-2041669517705388] Koenderink J. van Doorn A. Pinna B. Pepperell R. (2016) On right and wrong drawings. Art & Perception 4: 1–38.

[bibr9-2041669517705388] Koenderink J. J. Albertazzi L. van Doorn A. J. van Ee R. van de Grind W. A. Kappers A. M. de Vries S. (2010) Does monocular visual space contain planes? Acta Psychologica 134: 40–47. 20053390 10.1016/j.actpsy.2009.12.002

[bibr10-2041669517705388] Koenderink J. J. van Doorn A. J. Kappers A. M. Doumen M. J. Todd J. T. (2008) Exocentric pointing in depth. Vision Research 48: 716–723. 18221978 10.1016/j.visres.2007.12.002

[bibr11-2041669517705388] Koenderink J. J. van Doorn A. J. Lappin J. S. (2003) Exocentric pointing to opposite targets. Acta Psychologica 112: 71–87. 12423900 10.1016/s0001-6918(02)00101-4

[bibr12-2041669517705388] Koenderink J. J. van Doorn A. J. Todd J. T. (2009) Wide distribution of local sign in the normal population. Psychological Research 73: 14–22. 18386052 10.1007/s00426-008-0145-7

[bibr13-2041669517705388] Mercator G. (1569) Nova et Aucta Orbis Terrae Descriptio ad Usum Navigantium Emendata *[New and more complete representation of the terrestrial globe properly adapted for use in navigation]*, Antwerpen, Belgium: Plantijn.

[bibr14-2041669517705388] Phillips F. Voshell M. G. (2009) Distortions of posterior visual space. Perception 38: 1045–1052. 19764306 10.1068/p6166

[bibr15-2041669517705388] Polyak S. L. (1941) The retina, Chicago, IL: The University of Chicago Press.

[bibr16-2041669517705388] Reid T. (1819) An inquiry into the human mind: On the principles of common sense, Edinburgh, Scotland: Stirling & Slade.

[bibr17-2041669517705388] Ricoh. (2016). Ricoh. Retrieved from https://theta360.com/en/.

[bibr18-2041669517705388] Wagemans J. van Doorn A. J. Koenderink J. J. (2011) Measuring 3d point configurations in pictorial space. i-Perception 2: 77–111. 23145227 10.1068/i0420 PMC3485770

